# Durable immunovirological control after sequential mogamulizumab and tucidinostat in relapsed or refractory adult T-cell leukemia/lymphoma

**DOI:** 10.3389/fimmu.2026.1919613

**Published:** 2026-09-08

**Authors:** Tatsuro Jo, Kazuhiro Noguchi, Takahiro Sakai, Kaho Umemoto, Kaori Yamaguchi, Saori Ikegami, Rena Baba, Tomoya Inoue, Sadaharu Irie, Masatoshi Matsuo, Yasushi Sawayama, Jun Taguchi, Kuniko Abe, Kazuto Shigematsu, Yasushi Miyazaki

**Affiliations:** 1 Department of Hematology, Japanese Red Cross Nagasaki Genbaku Hospital, Nagasaki City, Nagasaki, Japan; 2 Department of Clinical Laboratory, Japanese Red Cross Nagasaki Genbaku Hospital, Nagasaki City, Nagasaki, Japan; 3 Department of Pharmacy, Japanese Red Cross Nagasaki Genbaku Hospital, Nagasaki City, Nagasaki, Japan; 4 Department of Pathology, Japanese Red Cross Nagasaki Genbaku Hospital, Nagasaki City, Nagasaki, Japan; 5 Department of Hematology, Nagasaki University Hospital, Nagasaki City, Nagasaki, Japan

**Keywords:** adult T-cell leukemia/lymphoma, ATLL, HTLV-1 proviral load, memory CD8+ T cell, mogamulizumab, Tax301–309-specific CD8+ T cell, T-cell receptor Vβ repertoire, tucidinostat

## Abstract

Adult T-cell leukemia/lymphoma (ATLL) is an aggressive HTLV-1-associated T-cell neoplasm, and durable control of relapsed/refractory disease remains difficult without allogeneic hematopoietic stem cell transplantation. Mogamulizumab, an afucosylated anti-C-C chemokine receptor type 4 (CCR4) monoclonal antibody, and tucidinostat, an oral histone deacetylase inhibitor, have activity in this setting, but the immunovirological consequences of sequential CCR4-directed therapy and epigenetic modulation are unclear. We describe two patients with relapsed or refractory ATLL who achieved prolonged disease control after mogamulizumab/tucidinostat-based sequential therapy and underwent longitudinal monitoring of Tax301–309-specific CD8+ T cells, CD4/CD8 dynamics, HTLV-1 proviral DNA, and T-cell receptor (TCR) Vβ repertoire. Patient 1 had lymphoma-type ATLL with metabolic partial response after mogamulizumab plus EPOCH; tucidinostat was initiated 63 days after the final mogamulizumab dose. Tax301–309-specific CD8+ T cells increased from 0.03% at tucidinostat initiation to >0.8% during long-term follow-up, and disease control persisted for 32 months after tucidinostat initiation. Patient 2 had multiply relapsed ATLL and received four cycles of sequential mogamulizumab/tucidinostat, followed by rapid disappearance of circulating ATLL cells, normalization of soluble interleukin-2 receptor, and marked reduction of HTLV-1 proviral load from 565.7 copies/1,000 peripheral blood mononuclear cells to low levels. Disease control persisted for 17 months after treatment discontinuation. In both patients, TCR Vβ repertoire analysis showed memory CD8+ T-cell-predominant patterns, including expansion of a Vβ2 memory CD8+ T-cell population. These observations are hypothesis-generating and suggest that sequential tumor debulking, CCR4/Treg-axis modulation, and epigenetic treatment may create a context permissive for HTLV-1 antigen-specific cellular immune surveillance in selected patients with ATLL.

## 1 Introduction

Adult T-cell leukemia/lymphoma (ATLL) is a mature T-cell malignancy etiologically linked to chronic infection with human T-lymphotropic virus type 1 (HTLV-1). Aggressive ATLL is characterized by chemotherapy resistance, recurrent relapse, immune dysfunction, and poor long-term survival (1–4). Intensive multi-agent chemotherapy, anti-C-C chemokine receptor type 4 (CCR4) therapy, epigenetic agents, and allogeneic hematopoietic stem cell transplantation (allo-HSCT) have improved outcomes in selected patients; however, many patients are elderly, medically frail, or ineligible for allo-HSCT. The curative potential of allo-HSCT nevertheless underscores a central biological principle: ATLL can be controlled by immune-mediated mechanisms when sufficiently effective antitumor immunity is established.

Mogamulizumab is an afucosylated humanized monoclonal antibody targeting CCR4, which is commonly expressed on ATLL cells (5, 6). Mogamulizumab monotherapy has demonstrated clinical activity in relapsed or refractory ATLL (7–11). In addition to direct elimination of CCR4-positive malignant cells through enhanced antibody-dependent cellular cytotoxicity, mogamulizumab can deplete CCR4-positive regulatory T cells (Tregs), thereby potentially remodeling immunosuppressive cellular networks (12). Tucidinostat (chidamide; HBI-8000) is an oral histone deacetylase (HDAC) inhibitor that has demonstrated clinical activity as monotherapy in relapsed or refractory ATLL (13–15). Valemetostat, a dual enhancer of zeste homolog 1/2 (EZH1/2) inhibitor, has also demonstrated clinically meaningful activity as monotherapy in relapsed or refractory ATLL (16), reinforcing the therapeutic relevance of epigenetic dependence in this disease. These agents indicate that ATLL is pharmacologically vulnerable to both immune-directed and epigenetic interventions, although neither approach reliably produces durable remission when used alone in advanced disease.

The biological rationale for integrating tumor debulking, epigenetic modulation, and immune monitoring is strengthened by the molecular complexity of ATLL. Integrated genomic and epigenomic studies have shown recurrent alterations affecting T-cell receptor/nuclear factor kappa B signaling, immune surveillance, transcriptional regulation, and chromatin states (17–19). Because these alterations may modify both tumor-intrinsic susceptibility and host immune recognition, longitudinal immunological assessment may be essential for interpreting unusually durable clinical courses.

HTLV-1-derived antigens provide a conceptual link between direct tumor reduction and host immune control. Tax is a dominant target of HTLV-1-specific CD8+ T cells, and Tax-specific CD8+ T cells have been implicated in immune control of HTLV-1-infected cells and ATLL (20–24). HBZ-specific T-cell responses have also been described, particularly after allo-HSCT (25). Although Tax expression may be low or intermittently repressed in established ATLL, Tax remains highly immunogenic, and preservation or restoration of Tax-specific CD8+ T-cell responses may be relevant to long-term disease control. We previously reported that long-term survivors of aggressive-type ATLL can maintain Tax-specific memory CD8+ T cells and that live attenuated varicella-zoster virus vaccination can activate Tax-specific CD8+ T cells in ATLL (23, 24).

Here, we report two patients with relapsed or refractory ATLL who experienced prolonged disease control after sequential mogamulizumab and tucidinostat-based therapy. We focus on longitudinal Tax301–309-specific CD8+ T cells, CD4/CD8 dynamics, HTLV-1 proviral load, and T-cell receptor (TCR) Vβ repertoire remodeling. The aim is not to infer therapeutic efficacy from two cases, but to describe a coherent immunovirological pattern that may inform future immune-monitoring studies in ATLL.

## 2 Materials and methods

### 2.1 Study design, ethics, and clinical assessment

This is a descriptive, longitudinal two-patient report based on patients with relapsed or refractory ATLL treated in routine clinical practice at the Japanese Red Cross Nagasaki Genbaku Hospital. Clinical subtype and response were assessed descriptively using ATLL/lymphoma clinical practice criteria, incorporating physical examination, peripheral-blood findings, laboratory markers, computed tomography (CT), and positron emission tomography/computed tomography (PET-CT) when available. Patient inclusion was not based on HLA genotype. Both patients were HLA-A*24:02-positive, which permitted longitudinal monitoring using the same HLA-A*24:02-restricted Tax301–309 tetramer assay. No formal statistical testing was performed because the study was exploratory and included only two patients. For Patient 2, available historical immune and virological monitoring data extending from the first relapse onward were also reviewed to provide within-patient longitudinal context for the immunovirological changes observed during the current treatment course.

The study was conducted in accordance with the Declaration of Helsinki and its later amendments, and approved by the institutional Ethical Review Board (approval number: R3-668; approval date: 29 March 2021). Written informed consent for publication and immune/virological monitoring was obtained from both patients.

### 2.2 Diagnostic procedure and clinical laboratory monitoring

ATLL diagnosis was based on clinical presentation, histopathology or peripheral-blood findings, HTLV-1 seropositivity, and confirmation of monoclonal HTLV-1 integration by Southern blotting when available. Immunohistochemistry for Patient 1 included T-cell lineage markers, CD30, CCR4, ALK, Epstein-Barr virus-encoded small RNA (EBER), T-cell intracellular antigen-1 (TIA-1), and granzyme B. Serial complete blood counts, differential counts, lactate dehydrogenase (LDH), soluble interleukin-2 receptor (sIL-2R), C-reactive protein, and other routine chemistry parameters were obtained as part of clinical care. In Patient 2, KL-6 and chest CT findings were monitored after the development of interstitial pneumonia.

### 2.3 Flow cytometric monitoring of T-cell subsets and Tax301–309-specific CD8+ T cells

Peripheral blood mononuclear cells (PBMCs) were serially analyzed by flow cytometry in the institutional clinical laboratory. CD3-positive, CD4-positive, and CD8-positive T-cell fractions were quantified among PBMCs using a consistent longitudinal gating strategy. Because both patients were human leukocyte antigen (HLA)-A*24:02-positive, HTLV-1 Tax301–309-specific CD8+ T cells were quantified using an HLA-A*24:02/HTLV-1 Tax301–309 tetramer containing the peptide SFHSLHLLF (MBL, Nagoya, Japan) (22, 26). Tetramer-positive CD8+ T cells were recorded longitudinally as Tax301–309-specific CD8+ T cells. Tetramer positivity was interpreted as peptide–MHC-specific T-cell receptor recognition and was not considered evidence of cellular cytotoxicity. Absolute counts of Tax301–309-specific CD8+ T cells were not systematically available; therefore, the analysis emphasized within-patient temporal changes rather than cross-sectional comparison between patients. A representative flow-cytometric gating strategy is shown in **Supplementary Figure 2**.

### 2.4 TCR Vβ repertoire and CD8+ T-cell subset analysis

TCR Vβ repertoire analysis was performed using a flow cytometric Vβ antibody panel covering multiple TCR Vβ families, according to our institutional immune-monitoring studies (23, 24). In the present study, TCR Vβ repertoire analysis was restricted to CD8+ T cells and was not performed in the CD4-positive T-cell compartment. CD8+ T cells were further evaluated using CD27/CD45RA-based phenotyping. Memory CD8+ T cells were defined as CD8+CD27+CD45RA− cells, effector CD8+ T cells as CD8+CD27−CD45RA+ cells, and naïve CD8+ T cells as CD8+CD27+CD45RA+ cells, according to our previously described approach (23). Vβ-family frequencies were assessed separately within effector and memory CD8+ T-cell compartments. A representative gating strategy is shown in **Supplementary Figure 2**. In this exploratory report, Vβ-family populations ≥10% were considered expanded and those ≥15% were considered markedly expanded. Because antibody-based Vβ analysis resolves family-level over-representation rather than sequence-level clonality, these populations are described as expanded or over-represented Vβ-family CD8+ T-cell populations, not as definitively clonal T-cell populations. Accordingly, Vβ2 memory CD8+ T-cell expansion was regarded as hypothesis-generating evidence of repertoire remodeling and not as proof that these cells were Tax301–309-specific, because tetramer-positive cells were not sorted for single-cell TCR sequencing or functional assays.

### 2.5 HTLV-1 proviral DNA analysis

HTLV-1 proviral DNA was quantified in PBMCs and expressed as copies per 1,000 PBMCs. Proviral-load interpretation was integrated with disease compartment. In blood-involved relapse, proviral DNA was interpreted together with circulating ATLL-cell counts and sIL-2R. In lymphoma-type disease with minimal circulating tumor cells, proviral DNA was interpreted more cautiously because it may reflect malignant cells, non-malignant HTLV-1-infected cells, or both.

## 3 Results

### 3.1 Patient 1: Tax301–309-specific CD8+ T-cell expansion after salvage tucidinostat following Moga-EPOCH

**Figure 1:**
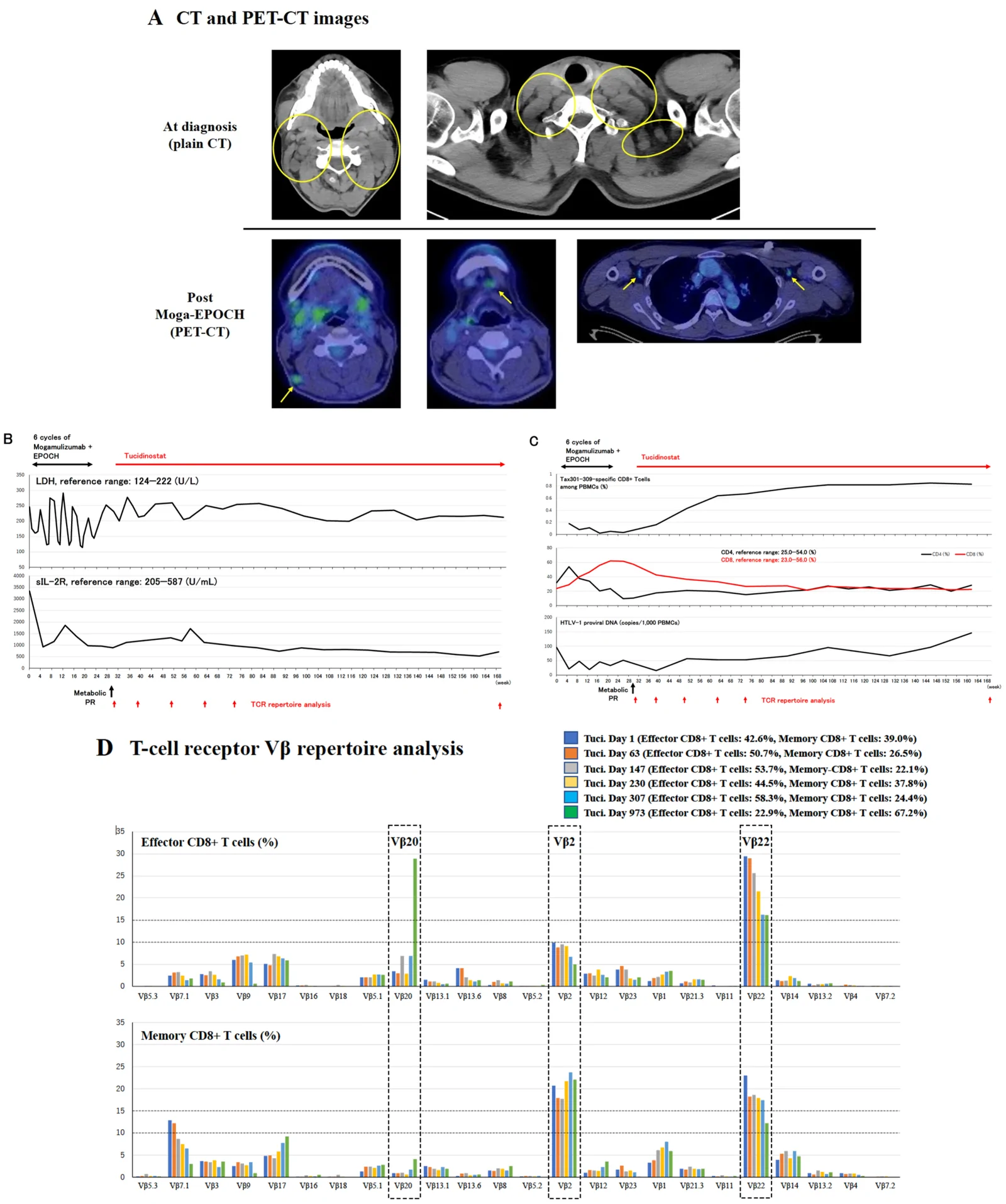
Clinical course and longitudinal immunovirological monitoring of Patient 1. **(A)** Representative computed tomography (CT) images at diagnosis and positron emission tomography/computed tomography (PET-CT) images after six cycles of mogamulizumab plus EPOCH (Moga-EPOCH), showing metabolic partial response. **(B)** Serial lactate dehydrogenase (LDH) and soluble interleukin-2 receptor (sIL-2R) levels during Moga-EPOCH and subsequent tucidinostat treatment. **(C)** Longitudinal frequency of Tax301–309-specific CD8+ T cells among peripheral blood mononuclear cells (PBMCs), serial CD4-positive and CD8-positive T-cell fractions among PBMCs, and serial HTLV-1 proviral DNA levels expressed as copies per 1,000 PBMCs. **(D)** Serial T-cell receptor (TCR) Vβ repertoire analysis of effector and memory CD8+ T-cell compartments after tucidinostat initiation. Vβ20, Vβ2, and Vβ22 are highlighted because they showed notable expansion or longitudinal changes during follow-up. Dashed horizontal lines indicate the 10% and 15% thresholds used to define expanded and markedly expanded Vβ-family CD8+ T-cell populations, respectively. Red arrowheads below panels B and C indicate the time points at which the TCR Vβ repertoire analyses shown in panel D were performed. The six sampling time points correspond to tucidinostat days 1, 63, 147, 230, 307, and 973. EPOCH, etoposide, prednisolone, vincristine, cyclophosphamide, and doxorubicin; HTLV-1, human T-lymphotropic virus type 1; LDH, lactate dehydrogenase; PBMCs, peripheral blood mononuclear cells; PET-CT, positron emission tomography/computed tomography; sIL-2R, soluble interleukin-2 receptor; TCR, T-cell receptor; Tuci, tucidinostat.

A 54-year-old man developed left submandibular and cervical swelling in January 2023. Left cervical lymph-node biopsy showed large atypical lymphoid cells positive for CD2, CD3, CD4, CD30, and CCR4 and negative for CD8, ALK, EBER, TIA-1, and granzyme B, consistent with peripheral T-cell lymphoma with features suggestive of an anaplastic large cell lymphoma-like ATLL phenotype (**Table 1**). At referral, peripheral-blood abnormal lymphocytes were not evident, and hematopoiesis was preserved, with hemoglobin 14.5 g/dL and platelet count 250 × 10^9^/L. LDH was 246 U/L and sIL-2R was 3,341 U/mL. CT demonstrated bilateral cervical and supraclavicular lymphadenopathy, suspected periportal hepatic infiltration, and splenomegaly (**Figure 1A**). HTLV-1 antibody was positive, and Southern blotting confirmed monoclonal HTLV-1 integration. He was diagnosed with lymphoma-type ATLL and did not undergo allo-HSCT according to the patient’s preference.

**Table 1:** Laboratory data at first diagnosis of Patient 1.

Parameter	Value
White blood cell (/µL)	7,200
Neutrophil (%)	70.5
Lymphocyte (%)	17.0
Abnormal lymphocyte (%)	1.0
Large granular lymphocyte (%)	2.5
Red blood cell (×10^6^/µL)	4.85
Hemoglobin (g/dL)	14.5
Hematocrit (%)	43.5
Platelet (×10^9^/L)	250
CD3 (%)	48.6
CD4 (%)	31.6
CD8 (%)	23.7
Lactate dehydrogenase (U/L)	246
C-reactive protein (mg/dL)	1.45
Soluble interleukin-2 receptor (U/mL)	3,341
HTLV-1 Ab (S/CO)	118.0
HTLV-1 proviral DNA (copies/1,000 PBMCs)	96.1
HTLV-1 monoclonal integration	Positive by Southern blotting
Pathological diagnosis	Peripheral T-cell lymphoma, suggestive of an anaplastic large cell lymphoma-like ATLL phenotype
Immunophenotype	CD2+, CD3+, CD4+, CD30+, CCR4+; CD8-, ALK-, EBER-, TIA-1-, granzyme B-

ALK, anaplastic lymphoma kinase; ATLL, adult T-cell leukemia/lymphoma; CCR4, C-C chemokine receptor type 4; EBER, Epstein-Barr virus-encoded small RNA; HTLV-1, human T-lymphotropic virus type 1; PBMCs, peripheral blood mononuclear cells; S/CO, signal-to-cutoff ratio; TIA-1, T-cell intracellular antigen-1.

Mogamulizumab plus EPOCH (Moga-EPOCH), a mogamulizumab-containing EPOCH regimen previously reported for newly diagnosed aggressive ATLL (27), was administered for six cycles from March to August 2023. Although tumor burden decreased, CT in September 2023 showed mild re-enlargement of submandibular and left axillary lymph nodes, and PET-CT in October 2023 indicated metabolic partial response rather than complete response (CR). Tucidinostat was started 63 days after the final mogamulizumab dose. Thereafter, LDH and sIL-2R gradually declined and remained largely within or near the reference range during follow-up (**Figure 1B**).

Tax301–309-specific CD8+ T cells decreased during Moga-EPOCH and reached 0.03% at tucidinostat initiation. After tucidinostat was introduced, Tax301–309-specific CD8+ T cells increased rapidly, exceeded 0.6% by approximately seven months, and remained >0.8% beyond 24 months (**Figure 1C**). The CD4/CD8 balance inverted during Moga-EPOCH, with CD8 predominance during and shortly after chemoimmunotherapy, followed by gradual recovery of the CD4 fraction during long-term tucidinostat treatment. HTLV-1 proviral DNA was not markedly elevated compared with blood-dominant relapse patterns and fluctuated without overt leukemic progression during follow-up (**Figure 1C**).

Serial TCR Vβ repertoire analysis from tucidinostat initiation showed expanded Vβ22 effector and memory CD8+ T-cell populations that persisted over time, although their relative frequencies declined. A Vβ2 memory CD8+ T-cell population was already prominent at tucidinostat initiation and remained detectable through day 973. Total effector CD8+ T cells exceeded total memory CD8+ T cells at tucidinostat initiation and day 307; by day 973, total memory CD8+ T cells exceeded total effector CD8+ T cells (67.2% vs 22.9%), consistent with a transition toward memory CD8+ T-cell predominance during sustained disease control (**Figure 1D**). A newly expanded Vβ20 effector CD8+ T-cell population was observed on day 973, but because it emerged during clinically controlled disease, its antigenic driver could not be inferred.

At the last follow-up, the patient remained under clinical disease control 32 months after tucidinostat initiation and 34 months after the final mogamulizumab dose.

### 3.2 Patient 2: rapid proviral-load reduction and memory CD8+ T-cell predominance after sequential mogamulizumab/tucidinostat

Patient 2 had been followed since January 2009 for chronic-type ATLL. In July 2011, at 66 years of age, bone marrow involvement developed and the disease transformed to acute-type ATLL. HTLV-1 monoclonal integration was documented. Four cycles of modified LSG15 (mLSG15) therapy induced CR, followed by observation. A first relapse occurred in January 2016 with 43% circulating ATLL cells, sIL-2R 5,590 U/mL, splenomegaly, and no lymphadenopathy; six cycles of mogamulizumab plus CHOP induced CR. A second relapse occurred in December 2018 with 44% circulating ATLL cells, sIL-2R 5,150 U/mL, and lymphadenopathy; six cycles of Moga-EPOCH again induced CR. Lenalidomide maintenance was attempted but discontinued after eight days because of rash.

Longitudinal immune and virological monitoring data were available from the first relapse onward (**Supplementary Figure 1**). Across the first, second, and third relapses, increases in the CD4-positive T-cell fraction and decreases in the CD8-positive T-cell fraction were accompanied by increases in HTLV-1 proviral DNA. Following the earlier mogamulizumab-containing chemoimmunotherapy courses, the CD4/CD8 balance inverted toward CD8 predominance and HTLV-1 proviral DNA markedly decreased. Frequencies of Tax301–309-specific CD8+ T cells also decreased around each relapse. Notably, the frequencies of Tax301–309-specific CD8+ T cells observed during this earlier longitudinal period remained lower than the higher frequencies subsequently observed after sequential mogamulizumab/tucidinostat therapy.

In October 2024, the patient developed a third relapse with white blood cell count 10,800/µL, 38% ATLL cells, sIL-2R 3,001 U/mL, mild lymphadenopathy, and mild splenomegaly (**Table 2**). Hemoglobin and platelet count remained preserved at 14.7 g/dL and 183 × 10^9^/L, respectively; the platelet count at first diagnosis had been 200 × 10^9^/L. Because of extensive prior exposure to cytotoxic agents and reduced left ventricular ejection fraction (47.9%), intensive chemotherapy was avoided. Sequential therapy was administered for four cycles: mogamulizumab was given first and then every four weeks, while tucidinostat was administered between mogamulizumab doses at 2- to 3-day intervals for six doses per cycle, with dose adjustment from 20 to 30 mg according to tolerability.

**Table 2:** Laboratory data of Patient 2 at first diagnosis and third relapse.

Parameter	First diagnosis	Third relapse
White blood cell (/µL)	12,400	10,800
Neutrophil (%)	51.0	40.0
Lymphocyte (%)	24.0	11.0
Abnormal lymphocyte (%)	16.0	38.0
Large granular lymphocyte (%)	0.0	3.0
Red blood cell (×10^6^/µL)	4.69	4.68
Hemoglobin (g/dL)	14.8	14.7
Platelet (×10^9^/L)	200	183
Hematocrit (%)	43.5	43.9
CD3 (%)	47.4	79.5
CD4 (%)	43.6	69.4
CD8 (%)	25.2	9.8
Lactate dehydrogenase (U/L)	174	234
C-reactive protein (mg/dL)	0.11	1.45
Soluble interleukin-2 receptor (U/mL)	1,390	3,001
HTLV-1 Ab (S/CO)	11.60	Not examined
HTLV-1 proviral DNA (copies/1,000 PBMCs)	Not examined	565.7
HTLV-1 monoclonal integration	Positive by Southern blotting	Not examined

ATLL, adult T-cell leukemia/lymphoma; HTLV-1, human T-lymphotropic virus type 1; PBMCs, peripheral blood mononuclear cells; S/CO, signal-to-cutoff ratio.

**Figure 2:**
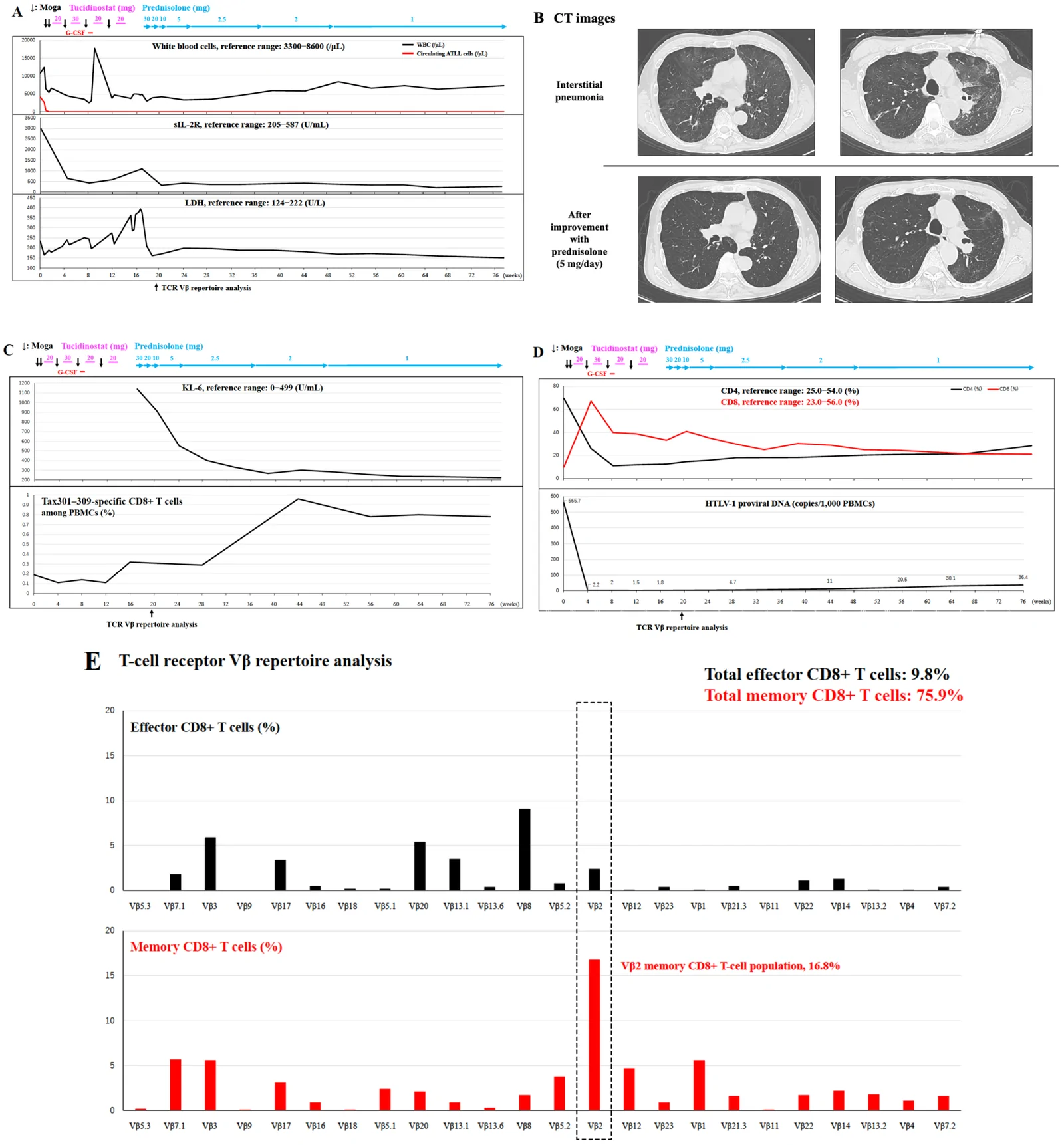
Clinical course and longitudinal immunovirological monitoring of Patient 2. **(A)** Serial white blood cell counts, circulating ATLL-cell counts, soluble interleukin-2 receptor (sIL-2R) levels, and lactate dehydrogenase (LDH) levels during sequential mogamulizumab/tucidinostat therapy. The rapid disappearance of circulating ATLL cells and normalization of sIL-2R demonstrate the hematologic response to treatment. The timing of mogamulizumab, tucidinostat, prednisolone, granulocyte colony-stimulating factor (G-CSF), and TCR Vβ repertoire analysis is shown. **(B)** Representative CT images at the diagnosis of interstitial pneumonia and after improvement with prednisolone treatment. **(C)** Serial KL-6 levels and the longitudinal frequency of Tax301–309-specific CD8+ T cells among PBMCs. The treatment schedule is shown above the graphs. **(D)** Serial CD4-positive and CD8-positive T-cell fractions among PBMCs and serial HTLV-1 proviral DNA levels expressed as copies per 1,000 PBMCs. The treatment schedule is shown above the graphs. **(E)** T-cell receptor (TCR) Vβ repertoire analysis after sequential mogamulizumab/tucidinostat therapy, showing memory CD8+ T-cell predominance and an expanded Vβ2 memory CD8+ T-cell population. Abbreviations: ATLL, adult T-cell leukemia/lymphoma; CT, computed tomography; G-CSF, granulocyte colony-stimulating factor; HTLV-1, human T-lymphotropic virus type 1; LDH, lactate dehydrogenase; Moga, mogamulizumab; PBMCs, peripheral blood mononuclear cells; sIL-2R, soluble interleukin-2 receptor; TCR, T-cell receptor.

Circulating ATLL cells disappeared rapidly after treatment initiation (**Figure 2A**). sIL-2R decreased and normalized (**Figure 2A**). Tucidinostat was initiated on October 25, 2024, and the final dose was administered on January 30, 2025. LDH increased during treatment and reached 395 U/L after the fourth cycle (**Figure 2A**). Because exertional dyspnea and fatigue developed, chest CT performed on February 2, 2025, showed bilateral diffuse ground-glass opacities, and KL-6 was 1,141 U/mL, leading to a diagnosis of drug-induced interstitial pneumonia (**Figures 2B,C**). No further tucidinostat was administered because of the interstitial pneumonia. Prednisolone 30 mg/day was initiated on February 14, 2025, for treatment of the interstitial pneumonia. Symptoms, CT findings, LDH, sIL-2R, and KL-6 subsequently improved during corticosteroid tapering. Interstitial pneumonia did not recur after tapering to low-dose prednisolone.

Tax301–309-specific CD8+ T cells remained <0.2% during sequential therapy but increased to 0.32% after four cycles (**Figure 2C**). During prednisolone 30 mg/day, the frequency remained stable at 0.29%; after tapering to 2.5 mg/day, Tax301–309-specific CD8+ T cells increased to 0.96% and subsequently remained around 0.8%. The CD4/CD8 balance was markedly CD4-predominant at third relapse (CD4 69.4%, CD8 9.8%), but inverted soon after treatment initiation, with prolonged CD8 predominance (**Figure 2D**). HTLV-1 proviral DNA decreased from 565.7 copies/1,000 PBMCs at relapse to low levels soon after treatment and remained markedly lower than the relapse baseline during follow-up, although low-level increases were observed later (**Figure 2D**).

TCR Vβ repertoire analysis after four cycles showed pronounced memory CD8+ T-cell predominance: total memory CD8+ T cells were 75.9%, whereas total effector CD8+ T cells accounted for 9.8%. A Vβ2 memory CD8+ T-cell population was markedly expanded to 16.8%, while no effector CD8+ T-cell population exceeded 10% (**Figure 2E**). These immunological findings coincided with disappearance of circulating ATLL cells, normalization of sIL-2R, marked reduction of proviral load, and sustained clinical disease control after treatment discontinuation.

At the last follow-up, the patient remained under clinical disease control 17 months after discontinuation of mogamulizumab/tucidinostat and 21 months after the third relapse.

## 4 Discussion

This Brief Research Report describes two patients with relapsed or refractory ATLL in whom durable disease control after sequential mogamulizumab and tucidinostat-based treatment was accompanied by longitudinal changes in HTLV-1-directed cellular immunity, CD4/CD8 balance, TCR repertoire, and, in blood-involved relapse, proviral burden. The central observation is not that a two-patient experience establishes a therapeutic regimen, but that disease control coincided with increased frequencies of Tax301–309-specific CD8+ T cells and memory CD8+ T-cell-predominant repertoire remodeling in a setting shaped by CCR4-directed therapy and epigenetic modulation. However, the sequence differed between the two patients: Patient 1 received tucidinostat after completion of Moga-EPOCH, whereas Patient 2 received repeated sequential mogamulizumab/tucidinostat cycles. Therefore, the shared feature of these cases should be interpreted as exposure to CCR4-directed therapy followed by or combined with epigenetic modulation, rather than as a uniform treatment regimen.

Several biological mechanisms may converge in this setting. Mogamulizumab can reduce CCR4-positive ATLL cells and, based on prior studies, may deplete CCR4-positive Tregs, potentially lowering tumor burden while relieving immunosuppressive pressure (7–12). In Patient 1, tucidinostat was initiated approximately two months after the final mogamulizumab dose, when the CD4-positive T-cell fraction was low. This timing raises the possibility that epigenetic modulation occurred in an immune environment already reshaped by CCR4-directed therapy. In Patient 2, repeated prior responses to mogamulizumab-containing therapy suggest retained vulnerability of CCR4-positive disease, while the addition of tucidinostat may have provided further direct or immune-modulatory pressure.

Valemetostat deserves explicit consideration because it represents a different, clinically validated epigenetic strategy in relapsed or refractory ATLL. The phase 2 study of valemetostat demonstrated that dual EZH1/2 inhibition can induce responses in this disease (16). Together with the activity of tucidinostat (13–15), these data support the view that ATLL is not only genetically complex but also epigenetically targetable. However, HDAC inhibition and EZH1/2 inhibition are mechanistically distinct: tucidinostat modifies histone acetylation and transcriptional programs, whereas valemetostat targets Polycomb-mediated chromatin repression. Therefore, the present findings should not be generalized to all epigenetic agents. A separate safety-related development involving tazemetostat, a selective EZH2 inhibitor used for follicular lymphoma and epithelioid sarcoma, merits only a limited comment: its voluntary market withdrawal after a signal of hematologic second primary malignancies highlights the need for long-term safety surveillance with epigenetic therapies, but this signal should not be directly extrapolated to valemetostat or to HDAC inhibition (28). Rather, the present data suggest that epigenetic therapy may be most informative when paired with immune and virological monitoring capable of distinguishing tumor debulking from immune-mediated disease control.

The interaction between HDAC inhibition and CCR4 biology should be interpreted cautiously. HDAC inhibitors can induce growth arrest and apoptosis in malignant lymphoid cells, modulate antigen presentation and immune susceptibility, and alter transcriptional states (29–31). Some experimental data suggest that HDAC inhibitors may downregulate CCR4 and reduce susceptibility to mogamulizumab in CCR4-positive mature T-cell lymphomas, whereas an ATLL case report showed that tucidinostat restored CCR4 expression and enabled mogamulizumab retreatment in a particular clinical context (32, 33). These apparently context-dependent observations support a sequence-dependent interpretation: timing, disease compartment, baseline epigenetic state, and prior CCR4-directed pressure may determine whether HDAC inhibition antagonizes or complements mogamulizumab-directed treatment.

Tax301–309-specific CD8+ T cells provide a plausible immune correlate of sustained control. Tax is highly immunogenic, and Tax-specific cellular immunity has been associated with control of HTLV-1-infected cells and long-term survival in ATLL (20–24). In both patients, Tax301–309-specific CD8+ T cells increased during the post-tucidinostat period in a clinical context shaped by preceding or concurrent mogamulizumab. However, because neither patient received tucidinostat monotherapy without preceding or concurrent mogamulizumab exposure in an immunologically comparable setting, the present observations cannot distinguish whether the increase in Tax301–309-specific CD8+ T cells was attributable to the sequential treatment strategy, to tucidinostat itself, or to other treatment-associated changes such as tumor reduction and immune reconstitution. In Patient 2, the delayed sharp expansion after corticosteroid tapering suggests that high-dose prednisolone may have transiently constrained Tax301–309-specific CD8+ T-cell expansion, although the underlying antigenic stimulus cannot be determined. These findings are compatible with re-emergence or amplification of pre-existing HTLV-1-specific CD8+ T-cell surveillance after tumor reduction and partial relief of immune suppression. The long-term historical data from Patient 2 provide additional within-patient context. Each relapse was accompanied by a shift toward CD4 predominance, increased HTLV-1 proviral burden, and decreased frequencies of Tax301–309-specific CD8+ T cells, whereas prior mogamulizumab-containing chemoimmunotherapy was followed by CD8 predominance and marked reduction of proviral DNA (**Supplementary Figure 1**). Moreover, frequencies of Tax301–309-specific CD8+ T cells during these earlier periods remained below the higher levels observed after sequential mogamulizumab/tucidinostat therapy. Although this recurrent pattern is consistent with an association between immunovirological status and clinical disease activity, it does not establish that expansion of Tax301–309-specific CD8+ T cells itself prevented relapse, particularly because the preceding treatment regimens and sampling intervals differed.

Both patients were HLA-A*24:02-positive, although HLA genotype was not a criterion for patient selection. HLA-A*24:02 is the most frequent HLA-A allele in the Japanese population and is carried by approximately 60% of individuals (34). Tax301–309 (SFHSLHLLF) is a well-characterized HLA-A*24:02-restricted HTLV-1 epitope (22, 26), and previous studies in ATLL have demonstrated that Tax301–309-specific CD8+ T cells can produce IFN-γ and exert cytotoxic activity against HLA-matched Tax-expressing ATLL cells (26). Thus, although cytotoxic function was not directly assessed in the present patients, the tetramer-positive cells observed here may include functionally competent Tax301–309-specific CD8+ T cells. Nevertheless, our findings cannot be generalized to patients with other HLA genotypes. Other well-characterized HLA-restricted Tax epitopes include HLA-A*02:01-restricted Tax11–19 (26) and HLA-A*11:01-restricted Tax88–96 and Tax272–280 (35), providing opportunities for analogous HLA-matched immune monitoring in future studies.

The TCR Vβ data provide complementary information on broader CD8+ T-cell repertoire remodeling beyond the single HLA-A*24:02-restricted Tax301–309 specificity measured by tetramer staining. Previous single-cell studies have demonstrated that HLA-A*24:02-restricted Tax301–309-specific CD8+ T cells can exhibit highly restricted TCR repertoires, including strongly biased BV7 usage and a conserved PDR motif in the TCR-β CDR3 region (22). In the present patients, however, the expanded Vβ2 memory populations were not isolated from Tax301–309 tetramer-positive cells and therefore cannot be assigned Tax301–309 specificity. These Vβ changes may instead reflect expansion of multiple CD8+ T-cell populations directed against Tax or other antigens and should be interpreted as evidence of broader repertoire remodeling rather than as a Tax-specific TCR signature. TCR Vβ staining has also been used to identify malignant clones in ATLL and to demonstrate autologous CD8+ T-cell-mediated killing in a mechanistic setting (36). Nevertheless, Vβ-family analysis is not sequence-level TCR sequencing, and the Vβ2 population was not sorted or functionally validated. Therefore, the present data should be framed as evidence of CD8+ T-cell repertoire remodeling rather than proof that Vβ2 CD8+ T cells are Tax301–309-specific or cytotoxic against ATLL cells. Future studies should combine Tax301–309 tetramer-guided sorting, single-cell TCR sequencing, HBZ peptide-stimulation assays, cytokine assays, and autologous tumor-cell killing assays.

The present observations also connect with our broader immune-monitoring work across hematological malignancies. In chronic-phase chronic myeloid leukemia, we reported that treatment-free remission after tyrosine kinase inhibitor discontinuation was associated with activated memory CD8+ T-cell populations and TCR Vβ skewing (37). In TFH-type lymphoma treated with denileukin diftitox, we observed that therapeutic perturbation of an immunosuppressive microenvironment was accompanied by CD8+ T-cell changes during clinical response (38). In relapsed or refractory large B-cell lymphoma and follicular lymphoma treated with epcoritamab, exploratory longitudinal monitoring suggested that effector/memory CD8+ T-cell balance and Vβ repertoire remodeling may accompany durable disease control or treatment discontinuation (39). The current ATLL report extends this framework to a virally driven mature T-cell malignancy in which Tax301–309-specific CD8+ T cells and HTLV-1 proviral DNA provide disease-specific immunovirological anchors.

The virological findings require compartment-aware interpretation. Patient 2 showed a dramatic decline in HTLV-1 proviral load after treatment, consistent with depletion of circulating malignant or infected CCR4-positive cells and subsequent maintenance of low-level proviral burden. Patient 1, by contrast, had lymphoma-type disease without substantial circulating tumor cells, and proviral DNA fluctuated rather than clearly declined. Thus, proviral load may be more informative in blood-involved disease than in predominantly nodal ATLL. In future studies, proviral load should be interpreted alongside clone-specific assays, tumor-cell quantification, and tissue disease burden.

This report has important limitations. It includes only two patients, treatment schedules differed, prior therapies were extensive, and causal attribution is not possible. In particular, the present study cannot determine whether the observed increase in Tax301–309-specific CD8+ T cells required preceding or concurrent mogamulizumab exposure or could also occur with tucidinostat monotherapy. In addition, no patients who experienced early relapse after a sufficiently comparable mogamulizumab/tucidinostat-based treatment strategy and underwent the same longitudinal immune monitoring were available for analysis. Therefore, the present study cannot determine whether expansion of Tax301–309-specific CD8+ T cells is specifically associated with durable disease control or whether similar changes may also occur in patients who subsequently experience early relapse. Tregs, absolute counts of Tax301–309-specific CD8+ T cells, and cytokine-producing CD8+ T cells were not serially quantified in a way that fully defines the immune landscape. Tax301–309-specific CD8+ T cells were quantified by peptide–MHC tetramer binding but were not assessed by functional assays of cytokine production or cellular cytotoxicity. TCR Vβ analysis provides family-level over-representation, not definitive clonality. Furthermore, because TCR Vβ repertoire analysis was not performed in the CD4-positive T-cell compartment, we could not assess longitudinal changes in the TCR repertoire of HTLV-1-infected or malignant CD4-positive cells. The influence of drug-induced interstitial pneumonia, treatment interruption, and corticosteroid tapering in Patient 2 cannot be separated from the effects of tumor reduction. In addition, given that prolonged exposure to epigenetic agents may raise late-toxicity questions, longer follow-up and prospective pharmacovigilance are required before any maintenance-like strategy can be proposed. These limitations are substantial and preclude claims of efficacy or mechanism.

Despite these constraints, the cases are biologically informative. Durable control of relapsed or refractory ATLL without allo-HSCT remains uncommon. The parallel observation of clinical control, expansion of Tax301–309-specific CD8+ T cells, memory CD8+ T-cell predominance, and marked proviral-load reduction in blood-involved relapse generates a testable model; sequential tumor debulking, CCR4/Treg-axis modulation, and epigenetic treatment may permit restoration of HTLV-1 antigen-specific cellular immune surveillance in selected patients. Prospective immune-monitoring studies that include both patients with durable disease control and those with early relapse are needed to determine whether expansion of Tax301–309-specific CD8+ T cells is specifically associated with durable remission and whether this pattern can predict treatment-free intervals or vulnerability to relapse.

## 5 Conclusion

Sequential mogamulizumab and tucidinostat-based therapy was associated with prolonged disease control in two patients with relapsed or refractory ATLL. Disease control coincided with expansion of Tax301–309-specific CD8+ T cells, memory CD8+ T-cell-predominant TCR Vβ repertoire patterns, and, in blood-involved relapse, marked reduction of HTLV-1 proviral load. These findings are hypothesis-generating and support prospective studies integrating HLA-A*24:02/Tax301–309 tetramer analysis, HBZ peptide-stimulation assays, Treg monitoring, single-cell sequencing, and functional CTL assays.

## Data Availability

The data supporting the findings of this study are available from the corresponding author upon reasonable request. The data are not publicly available because of privacy and ethical restrictions.

## References

[1] Shimoyama M . Diagnostic criteria and classification of clinical subtypes of adult T-cell leukaemia-lymphoma. A report from the Lymphoma Study Group (1984-87). Br J Haematol. (1991) 79:428–37. doi: 10.1111/j.1365-2141.1991.tb08051.x 1751370

[2] Cook LB Fuji S Hermine O Bazarbachi A Ramos JC Ratner L . Revised adult T-cell leukemia-lymphoma international consensus meeting report. J Clin Oncol. (2019) 37:677–87. doi: 10.1200/JCO.18.00501 30657736 PMC6494249

[3] Katsuya H Ishitsuka K Utsunomiya A Hanada S Eto T Moriuchi Y . Treatment and survival among 1594 patients with ATL. Blood. (2015) 126:2570–7. doi: 10.1182/blood-2015-03-632489 26361794

[4] Tsukasaki K Utsunomiya A Fukuda H Shibata T Fukushima T Takatsuka Y . VCAP-AMP-VECP compared with biweekly CHOP for adult T-cell leukemia-lymphoma: Japan Clinical Oncology Group Study JCOG9801. J Clin Oncol. (2007) 25:5458–64. doi: 10.1200/JCO.2007.11.9958 17968021

[5] Yoshie O Fujisawa R Nakayama T Harasawa H Tago H Izawa D . Frequent expression of CCR4 in adult T-cell leukemia and human T-cell leukemia virus type 1-transformed T cells. Blood. (2002) 99:1505–11. doi: 10.1182/blood.V99.5.1505 11861261

[6] Ishida T Utsunomiya A Iida S Inagaki H Takatsuka Y Kusumoto S . Clinical significance of CCR4 expression in adult T-cell leukemia/lymphoma: its close association with skin involvement and unfavorable outcome. Clin Cancer Res. (2003) 9:3625–34. 14506150

[7] Yamamoto K Utsunomiya A Tobinai K Tsukasaki K Uike N Uozumi K . Phase I study of KW-0761, a defucosylated humanized anti-CCR4 antibody, in relapsed adult T-cell leukemia-lymphoma and peripheral T-cell lymphoma. J Clin Oncol. (2010) 28:1591–8. doi: 10.1200/JCO.2009.25.3575 20177026

[8] Ishida T Joh T Uike N Yamamoto K Utsunomiya A Yoshida S . Defucosylated anti-CCR4 monoclonal antibody (KW-0761) for relapsed adult T-cell leukemia-lymphoma: a multicenter phase II study. J Clin Oncol. (2012) 30:837–42. doi: 10.1200/JCO.2011.37.3472 22312108

[9] Ishida T Utsunomiya A Jo T Yamamoto K Kato K Yoshida S . Mogamulizumab for relapsed adult T-cell leukemia-lymphoma: updated follow-up analysis of phase I and II studies. Cancer Sci. (2017) 108:2022–9. doi: 10.1111/cas.13343 28776876 PMC5623751

[10] Yonekura K Kusumoto S Choi I Nakano N Ito A Suehiro Y . Mogamulizumab for adult T-cell leukemia-lymphoma: a multicenter prospective observational study. Blood Adv. (2020) 4:5133–45. doi: 10.1182/bloodadvances.2020003053 33091125 PMC7594399

[11] Cook LBM Demontis MA Sagawe S Witkover A Melamed A Turpin J . Molecular remissions are observed in chronic adult T-cell leukemia/lymphoma in patients treated with mogamulizumab. Haematologica. (2019) 104:e566–9. doi: 10.3324/haematol.2019.219253 PMC6959161 31004017

[12] Ni X Langridge T Duvic M . Depletion of regulatory T cells by targeting CC chemokine receptor type 4 with mogamulizumab. Oncoimmunology. (2015) 4:e1011524. doi: 10.1080/2162402X.2015.1011524 26140234 PMC4485778

[13] Utsunomiya A Izutsu K Jo T Yoshida S Tsukasaki K Ando K . Oral histone deacetylase inhibitor tucidinostat (HBI-8000) in patients with relapsed or refractory adult T-cell leukemia/lymphoma: phase IIb results. Cancer Sci. (2022) 113:2778–87. doi: 10.1111/cas.15431 35579212 PMC9357668

[14] Rai S Kim WS Ando K Choi I Izutsu K Tsukamoto N . Oral HDAC inhibitor tucidinostat in patients with relapsed or refractory peripheral T-cell lymphoma: phase IIb results. Haematologica. (2023) 108:811–21. doi: 10.3324/haematol.2022.280996 36200417 PMC9973490

[15] Kamiunten A Kameda T Sekine M Kawano H Toyama T Akizuki K . Effects of tucidinostat in adult T-cell leukemia/lymphoma in clinical practice. Int J Hematol. (2025) 122:83–92. doi: 10.1007/s12185-025-03963-9 40067419 PMC12202688

[16] Izutsu K Makita S Nosaka K Yoshimitsu M Utsunomiya A Kusumoto S . An open-label, single-arm phase 2 trial of valemetostat for relapsed or refractory adult T-cell leukemia/lymphoma. Blood. (2023) 141:1159–68. doi: 10.1182/blood.2022016862 36150143 PMC10651775

[17] Kataoka K Nagata Y Kitanaka A Shiraishi Y Shimamura T Yasunaga JI . Integrated molecular analysis of adult T cell leukemia/lymphoma. Nat Genet. (2015) 47:1304–15. doi: 10.1038/ng.3415 26437031

[18] Kogure Y Kameda T Koya J Yoshimitsu M Nosaka K Yasunaga JI . Whole-genome landscape of adult T-cell leukemia/lymphoma. Blood. (2022) 139:967–82. doi: 10.1182/blood.2021013568 34695199 PMC8854674

[19] Yamagishi M Kubokawa M Kuze Y Suzuki A Yokomizo A Kobayashi S . Chronological genome and single-cell transcriptome integration characterizes the evolutionary process of adult T-cell leukemia-lymphoma. Nat Commun. (2021) 12:4821. doi: 10.1038/s41467-021-25101-9 34376672 PMC8355240

[20] Kannagi M Harashima N Kurihara K Ohashi T Utsunomiya A Tanosaki R . Tumor immunity against adult T-cell leukemia. Cancer Sci. (2005) 96:249–55. doi: 10.1111/j.1349-7006.2005.00044.x 15904464 PMC11158966

[21] Bangham CRM . CTL quality and the control of human retroviral infections. Eur J Immunol. (2009) 39:1700–12. doi: 10.1002/eji.200939451 19582737

[22] Ishihara Y Tanaka Y Kobayashi S Kawamura K Nakasone H Gomyo A . A unique T-cell receptor amino acid sequence selected by human T-cell lymphotropic virus type 1 Tax301-309-specific cytotoxic T cells in HLA-A24:02-positive asymptomatic carriers and adult T-cell leukemia/lymphoma patients. J Virol. (2017) 91:e00974-17. doi: 10.1128/JVI.00974-17 28724766 PMC5599769

[23] Jo T Noguchi K Sakai T Kubota-Koketsu R Irie S Matsuo M . HTLV-1 Tax-specific memory cytotoxic T lymphocytes in long-term survivors of aggressive-type adult T-cell leukemia/lymphoma. Cancer Med. (2022) 11:3238–50. doi: 10.1002/cam4.4689 35315593 PMC9468428

[24] Jo T Kubota-Koketsu R Kaneko Y Sakai T Noguchi K Irie S . Live attenuated VZV vaccination induces antitumor immunity in ATLL patients. Cancer Immunol Immunother. (2023) 72:929–44. doi: 10.1007/s00262-022-03301-6 36181532 PMC10025209

[25] Narita T Ishida T Masaki A Suzuki S Ito A Mori F . HTLV-1 bZIP factor-specific CD4 T cell responses in adult T cell leukemia/lymphoma patients after allogeneic hematopoietic stem cell transplantation. J Immunol. (2014) 192:940–7. doi: 10.4049/jimmunol.1301952 24363428

[26] Suzuki S Masaki A Ishida T Ito A Mori F Sato F . Tax is a potential molecular target for immunotherapy of adult T-cell leukemia/lymphoma. Cancer Sci. (2012) 103:1764–73. doi: 10.1111/j.1349-7006.2012.02371.x 22735080 PMC7659279

[27] Jo T Matsuzaka K Shioya H Tominaga H Sakai T Kaneko Y . Mogamulizumab plus EPOCH therapy for patients with newly diagnosed aggressive adult T-cell leukemia/lymphoma. Anticancer Res. (2020) 40:5237–43. doi: 10.21873/anticanres.14527 32878812

[28] U.S. Food and Drug Administration . FDA alerts health care providers and patients about increased risk of new blood cancers with Tazverik (2026). Available online at: https://www.fda.gov/drugs/drug-alerts-and-statements/fda-alerts-health-care-providers-and-patients-about-increased-risk-new-blood-cancers-tazverik (Accessed June 16, 2026).

[29] West AC Johnstone RW . New and emerging HDAC inhibitors for cancer treatment. J Clin Invest. (2014) 124:30–9. doi: 10.1172/JCI69738 24382387 PMC3871231

[30] Seto E Yoshida M . Erasers of histone acetylation: the histone deacetylase enzymes. Cold Spring Harb Perspect Biol. (2014) 6:a018713. doi: 10.1101/cshperspect.a018713 24691964 PMC3970420

[31] Gameiro SR Malamas AS Tsang KY Ferrone S Hodge JW . Inhibitors of histone deacetylase 1 reverse the immune evasion phenotype to enhance T-cell mediated lysis of prostate and breast carcinoma cells. Oncotarget. (2016) 7:7390–7402. doi: 10.18632/oncotarget.7180 26862729 PMC4884926

[32] Kitadate A Ikeda S Abe F Takahashi N Shimizu N Matsue K . Histone deacetylase inhibitors downregulate CCR4 expression and decrease mogamulizumab efficacy in CCR4-positive mature T-cell lymphomas. Haematologica. (2018) 103:126–35. doi: 10.3324/haematol.2017.175646 29025909 PMC5777200

[33] Kawata T Shimizu T Shindo T Fujiwara K Morimoto S Watanabe M . Tucidinostat restores CCR4 expression in adult T-cell leukemia/lymphoma. Haematologica. (2024) 109:1007–9. doi: 10.3324/haematol.2023.283266 37794797 PMC10905079

[34] Ikeda N Kojima H Nishikawa M Hayashi K Futagami T Tsujino T . Determination of HLA-A, -C, -B, -DRB1 allele and haplotype frequency in Japanese population based on family study. Tissue Antigens. (2015) 85:252–9. doi: 10.1111/tan.12536 25789826 PMC5054903

[35] Harashima N Tanosaki R Shimizu Y Kurihara K Masuda T Okamura J . Identification of two new HLA-A*1101-restricted Tax epitopes recognized by cytotoxic T lymphocytes in an adult T-cell leukemia patient after hematopoietic stem cell transplantation. J Virol. (2005) 79:10088–92. doi: 10.1128/JVI.79.15.10088-10092.2005 16014972 PMC1181560

[36] Rowan AG Witkover A Melamed A Tanaka Y Cook LBM Fields P . T-cell receptor Vβ staining identifies the malignant clone in adult T-cell leukemia and reveals killing of leukemia cells by autologous CD8+ T cells. PLoS Pathog. (2016) 12:e1006030. doi: 10.1371/journal.ppat.1006030 27893842 PMC5125714

[37] Jo T Saburi Y Masunari T Noguchi K Sakai T Taguchi J . Activated memory cytotoxic T-lymphocytes and T-cell receptor Vβ clonality predict treatment-free remission after tyrosine kinase inhibitor discontinuation in chronic-phase chronic myeloid leukemia: a 1-year prospective immuno-monitoring study. Int J Mol Sci. (2026) 27:2713. doi: 10.3390/ijms27062713 41898572 PMC13026259

[38] Jo T Sakai T Noguchi K Yamaguchi K Umemoto K Matsuo M . Immune correlates of denileukin diftitox treatment in TFH-type lymphoma. Cancers. (2026) 18:1529. doi: 10.3390/cancers18101529 42192890 PMC13204862

[39] Jo T Taguchi J Sawayama Y Matsuo M Umemoto K Yamaguchi K . Clinical outcomes and exploratory longitudinal CTL/Vβ repertoire remodeling in patients with relapsed or refractory large B-cell lymphoma and follicular lymphoma treated with epcoritamab. Int J Mol Sci. (2026) 27:5132. doi: 10.3390/ijms27115132 42278654 PMC13257122

